# Unmasking the Enigma: Influenza Vaccine and the Rare Case of Post-Vaccination Pericarditis

**DOI:** 10.1155/2024/2729208

**Published:** 2024-03-09

**Authors:** Ekrem Yetiskul, Alaukika Agarwal, Gaetano Di Pietro, Faris Qaqish, Salman Khan, Shahkar Khan

**Affiliations:** Department of Internal Medicine, Staten Island University Hospital, 475 Seaview Avenue, Staten Island, NY 10305, USA

## Abstract

Acute pericarditis is an inflammatory condition involving the pericardium, the double-layered sac that surrounds the heart. It is characterized by chest pain, typically pleuritic and sharp, along with other clinical and laboratory findings indicative of pericardial inflammation. While acute pericarditis following influenza vaccination is rare, it has been reported in medical literature. The relationship between vaccinations, including the influenza vaccine, and pericarditis is particularly interesting, as it has implications for public health and vaccination programs. Understanding the pathophysiological mechanisms behind vaccine-induced pericarditis and recognizing the clinical presentation are essential for healthcare professionals to diagnose, manage, and educate patients appropriately.

## 1. Introduction

Influenza, an annually recurring global health concern, is a contagious respiratory infection caused by the influenza virus. To combat its widespread impact, routine influenza vaccination has proven to be an effective preventive measure. It is crucial, however, to acknowledge that, like all medical interventions, influenza vaccination is associated with potential side effects. One relatively rare side effect of influenza vaccination is pericarditis [1], an inflammatory condition of the pericardium of the heart. The incidence of hospital admissions of pericarditis is approximately 3.32 cases per 100,000 person-years [2]. This highlights the low incidence of the disease, despite the estimated incidence being considerably higher at 27.7 cases per 100,000 subjects per year [2, 3]. The incidence of pericarditis following influenza vaccination has not been reported in the existing literature. It is plausible that due to the low incidence of the disease, several cases of post-vaccination pericarditis are underreported [2, 3]. This case report describes a 91-year-old female who exhibited typical symptoms of acute pericarditis three days after receiving an influenza vaccine.

## 2. Case Presentation

**Figure 1:**
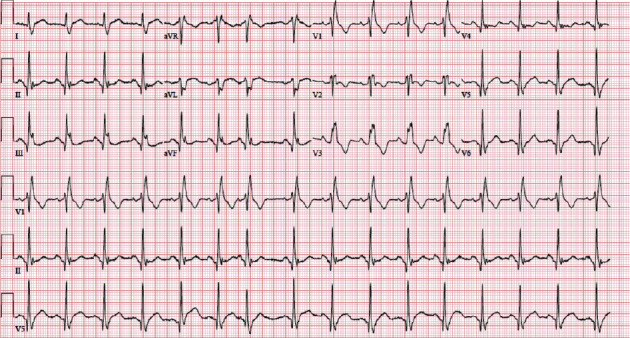
ECG obtained upon arrival to the emergency department with no ischemic changes.

**Figure 2:**
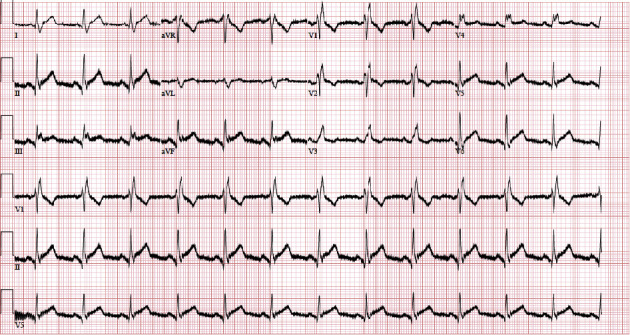
Subsequent ECG obtained 14 hours after initial ECG as shown in Figure 1. Subsequent ECG demonstrating ST elevations in inferior leads with PR depressions.

**Figure 3:**
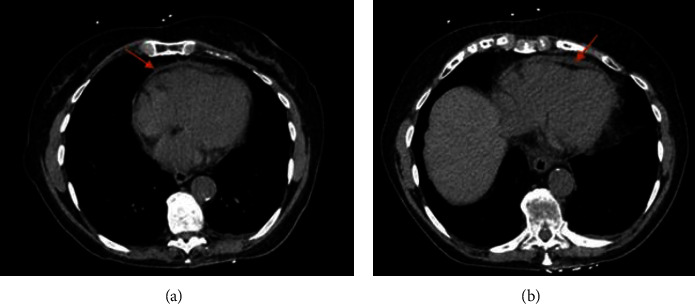
Computed tomography angiography with intravenous contrast of the chest demonstrating trace pericardial effusion (red arrows).

We present a case of a 91-year-old female with a past medical history of hypertension, hyperlipidemia, and presbycusis who presented to the emergency department complaining of chest pain, with sudden onset 2 hours prior to presentation. She described the chest pain as central, radiating to the left side of the chest, back, and left shoulder, and worse with inspiration. The patient also reported a low-grade fever, fatigue, and malaise. Notably, the patient received an inactivated influenza vaccine two days before the onset of symptoms. She denied any significant cardiac history, which was corroborated by her primary provider. On presentation to the ED, she was hypertensive with a blood pressure of (186/94 mmHg), tachycardic with a heart rate of 101 beats per minute (BPM), febrile (*T*_max_ 100.8 F), and saturating 99% on ambient room air. Upon examination, a pericardial friction rub was noted on auscultation. Laboratory values on admission were significant for leukocytosis with a white blood cell count (WBC) of 13.4 k K/uL, erythrocyte sedimentation rate (ESR) of 54 mm/hr, and C-reactive protein (CRP) of 227.9 mg/L. D-dimer and serial troponin markers were negative upon admission (Table 1). The initial electrocardiogram (ECG) showed no ischemic changes (Figure 1). Subsequent ECG performed 14 hours after the initial ECG showed ST elevations in inferior leads with PR depressions (Figure 2). Computed tomography angiography with intravenous contrast of the chest and aorta showed no evidence of intramural hematoma or dissection with atherosclerotic disease at the origins of the celiac axis, superior mesenteric, and renal arteries. A trace pericardial effusion was also present (Figure 3). A subsequent transthoracic echocardiogram was performed, which did not demonstrate any evidence of pericardial effusion. The patient's clinical presentation, including chest pain, fever, and the temporal association of these symptoms with the influenza vaccine, led to a preliminary diagnosis of acute pericarditis. The patient was admitted for observation and received ibuprofen and colchicine to relieve pain and reduce inflammation. The patient was advised to refrain from strenuous physical activity and was closely monitored for any worsening of symptoms. Her chest pain resolved over the next several days, and the fever subsided.

## 3. Discussion

Pericarditis is the inflammation of the pericardium, which is a sac-like cavity that encases the heart; however, etiology is not established in 90% of pericarditis cases [3, 4]. This may be due to an infectious or noninfectious cause. The diagnosis is largely clinical by presentation. It is more likely that males are hospitalized for acute pericarditis [5]. Risk factors indicating poor prognosis include temperature >100.4^0^F at presentation, subacute course, presence of tamponade, and resistance to NSAID treatment [6]. These symptoms may recur, which has been observed within 18 months of initial presentation in 30% of patients [7]. Routine laboratory values may reveal an elevated WBC count, ESR and CRP, which are nonspecific markers highlighting inflammation or infection in the body [8]. Additionally, cardiac enzymes such as troponins may be elevated [8, 9]. Colchicine is one drug that is effective in preventing both primary and recurrent pericarditis [9]. The use of colchicine along with NSAIDs has been shown to significantly reduce recurrences of this disease in patients with multiple recurrences [10].

Our case illustrates the occurrence of acute pericarditis following influenza vaccination in an elderly patient, underscoring the importance of vigilance in monitoring post-vaccination symptoms. While vaccine-induced pericarditis is rare, early recognition and appropriate management are essential to prevent complications and optimize patient outcomes.

Upon literature review, Mei et al. highlight a case report which serves as a crucial reminder of the potential rare adverse effects associated with influenza vaccination. They document an 87-year-old patient developing pericarditis post-influenza vaccination. This particular patient did not exhibit ECG abnormalities or elevated troponin levels, which makes this case even more noteworthy, as it challenges the conventional diagnostic markers of pericarditis [1].

Mei and colleagues' case is not an isolated incident, as they also highlight seven other patients with either a possible or well-defined association between influenza vaccination and pericarditis. This collective body of evidence underscores the need for clinicians and healthcare providers to remain alert to the potential link between pericarditis and influenza vaccination [1].

Moreover, an extensive review of the existing literature reveals a consistent trend in the rapid onset of pericarditis within a week of influenza vaccination. Notably, Meester, Luwaert, and Chaudron present two cases in which patients developed benign pericarditis within this timeframe [11]. In a different case, a 40-year-old male is found to have the onset of acute benign pericarditis within five days of the influenza vaccination [12]. The temporal correlation between vaccination and the onset of pericarditis observed in these cases suggests a causal relationship. Additionally, there has been one reported case of acute hemorrhagic pericarditis, which required surgical pericardiectomy followed by colchicine treatment [13].

Intriguingly, Streifler et al. present an even more unique case in which a patient experienced pericarditis after receiving the vaccine on two distinct occasions. This recurrence strongly implies a cause-and-effect relationship between the influenza vaccine and pericarditis [14]. Although such cases remain rare, they emphasize the importance of recognizing and managing vaccine-induced pericarditis early to prevent complications and optimize patient outcomes.

It is not necessary for acute symptomatic pericarditis to be the standalone presentation after vaccination. This may co-exist along with the exacerbation of Guillain–Barrè syndrome or nephrotic syndrome [15]. Kao et al. illustrate two cases and discuss this unique association between Guillain–Barrè syndrome, pericarditis, and nephrotic syndrome after influenza vaccination. This further implicates the administration of influenza vaccine with a higher risk of complications–not merely limited to one presentation at a time.

It is encouraging to note that most reported cases in the literature exhibit a favorable prognosis, with pericarditis typically resolving with anti-inflammatory therapy. However, a caveat exists for patients with a history of pericarditis. Their susceptibility to recurrent pericarditis following subsequent influenza vaccinations should be meticulously considered. The immunization history of such patients becomes a pivotal aspect of their medical record, particularly when chest pain is their chief complaint. Therefore, any decision to administer further vaccinations must be a collaborative process involving both the patient and their healthcare provider. This collaborative approach ensures that patients are adequately informed of the potential risks and benefits, allowing them to make informed decisions.

## 4. Conclusion

In summation, while pericarditis remains a rare side effect of influenza vaccination, the existence of recurrent cases in the literature emphasizes the importance of a patient's medical history, especially in instances where pericarditis is a prior diagnosis. Our case report illustrates a 91-year-old-female who presented with symptoms of acute pericarditis with significant elevations in inflammatory markers three days after receiving influenza vaccine. Her chest pain gradually resolved with colchicine and NSAID treatment. This case is essential in enhancing our understanding of potential side effects of vaccination and contributes to the ongoing discourse surrounding influenza vaccine safety. It is incumbent upon healthcare providers to engage patients in informed discussions regarding the influenza vaccine and any associated concerns. Vigilant monitoring of post-vaccination symptoms is equally vital to facilitate the early recognition and appropriate management of pericarditis, thereby ensuring the best possible outcomes for patients. This multifaceted approach to managing vaccine-induced pericarditis emphasizes the evolving landscape of vaccine safety and the ongoing need for healthcare professionals to remain informed and responsive to emerging clinical data.

## Figures and Tables

**Table 1:** Laboratory values at initial presentation.

	At initial presentation	Reference range
WBC	13.38 K/uL	4.8–10.8 K/uL
RBC	4.11 M/uL	4.20–5.40 M/uL
Hgb	13.2 g/dL	12.0–16.0 g/dL
PLT	201 K/uL	130–400 K/uL
Sodium	130 mmol/L	135–146 mmol/L
Potassium	5.3 mmol/L (hemolyzed)	3.5–5.0 mmol/L
Chloride	91 mmol/L	98–110 mmol/L
Anion gap	12 mmol/L	7–14 mmol/L
Bun	22 mg/dL	10–20 mg/dL
Cr	1 mg/dL	0.7–1.5 mg/dL
Glucose	120 mg/dL	70–99 mg/dL
Calcium	9.9 mg/dL	8.4–10.5 mg/dL
Protein total	6.7 g/dL	6.0–8.0 g/dL
Albumin	4.3 g/dL	3.5–5.2 g/dL
Bilirubin total	0.8 mg/dL	0.2–1.2 mg/dL
Alkaline phosphatase	56 U/L (hemolyzed)	30–115 U/L
AST	45 U/L (hemolyzed)	0–41 U/L
ALT	28 U/L (hemolyzed)	0–41 U/L
eGFR	53 mL/min/1.73 m^2^	≥60 mL/min/1.73m2
D dimer	<150 ng/mL DDU	≤229 ng/mL DDU
Troponin	<0.01 ng/mL	≤0.01 ng/mL
ESR	54 mm/Hr	0–20 mm/Hr
CRP	227.9 mg/L	≤4 mg/L

## Data Availability

The data used to support the findings of this study are available upon corresponding author's request.
